# Impact of a Novel Oblique-Tip Papillotome for Biliary Cannulation during ERCP: A Nonrandomized Coarsened Exact Matching Study

**DOI:** 10.1155/2020/2417841

**Published:** 2020-05-07

**Authors:** Hiroo Imazu, Shiaw-Hooi Ho, Shoryoku Hino, Khean-Lee Goh, Mitsuhiko Moriyama, Kazuki Sumiyama, Hisao Tajiri

**Affiliations:** ^1^Division of Gastroenterology and Hepatology, Department of Medicine, Nihon University School of Medicine, Tokyo, Japan; ^2^Department of Endoscopy, The Jikei University School of Medicine, Tokyo, Japan; ^3^Division of Gastroenterology, Department of Medicine, University of Malaya Medical Centre, Kuala Lumpur, Malaysia; ^4^Department of Neuropsychiatry, Ishikawa Prefecture Takamatsu Hospital, Kahoku, Japan; ^5^Department of Innovative Interventional Endoscopy Research, The Jikei University School of Medicine, Tokyo, Japan

## Abstract

**Background:**

We developed a novel oblique-tip papillotome (OT-papillotome) to facilitate biliary cannulation during endoscopic retrograde cholangiopancreatography (ERCP). This study was performed to evaluate the utility of the OT-papillotome for contrast-guided cannulation (CGC) and wire-guided cannulation (WGC) during ERCP, compared with standard cannulation by WGC using a standard-tip papillotome (ST-papillotome).

**Methods:**

A prospective study was performed at two centers. CGC with the OT-papillotome (OT-CGC group) was performed at Jikei University Hospital, while WGC was done with the OT-papillotome and ST-papillotome (OT-WGC and ST-WGC groups, respectively) at the University of Malaya Medical Centre. The results of the OT-CGC and OT-WGC groups were compared with those of the ST-WGC group after performing coarsened exact matching (CEM) to reduce bias due to nonrandomized and center-based patient allocation.

**Results:**

Eighty patients were enrolled in each of the OT-CGC, OT-WGC, and ST-WGC groups. After CEM, the successful biliary cannulation rate was significantly higher in the OT-CGC and OT-WGC groups than in the ST-WGC group, while rescue cannulation was reduced. The mean number of unintended pancreatic access events in the OT-WGC and OT-CGC groups was similar to the ST-WGC group. However, it was significantly lower in the OT-WGC group than in the OT-CGC group. Multivariate analysis revealed that the OT-papillotome was independently associated with less frequent rescue cannulation and a higher successful biliary cannulation rate.

**Conclusions:**

Although use of the OT-papillotome in biliary cannulation did not reduce unintended pancreatic access events or PEP compared to the ST-papillotome, the OT-papillotome increased the successful biliary cannulation rate, while reducing the frequency of rescue cannulation procedures. Combining the OT-papillotome with WGC might be the best cannulation technique for minimizing unintended pancreatic access.

## 1. Introduction

Various procedures related to endoscopic retrograde cholangiopancreatography (ERCP), including endoscopic biliary drainage, endoscopic choledocholithotomy, and per-oral cholangiopancreatoscopy, are essential for the diagnosis and treatment of pancreaticobiliary diseases. Post-ERCP pancreatitis (PEP) is the most common adverse event after ERCP-related procedures, and it has the potential to cause clinically significant morbidity and mortality [1–7]. Biliary cannulation is the first step of diagnostic and therapeutic ERCP, and cannulation-related factors such as difficulty with cannulation and unintended pancreatic access are thought to be associated with an increased risk of PEP [1–7]. Difficulty with cannulation may result in PEP due to papillary trauma, while unintended pancreatic access (such as contrast injection or advancing a guide wire into the pancreatic duct) can also cause PEP due to chemical and mechanical injury of the pancreas [8, 9]. It was also reported that unintended pancreatic access is significantly more frequent when cannulation is found to be difficult [7]. Therefore, it would seem logical that techniques and devices facilitating selective biliary cannulation could decrease the risk of PEP by minimizing papillary trauma and reducing unintended pancreatic duct access.

Two major cannulation techniques, wire-guided cannulation (WGC) and contrast-guided cannulation (CGC), can be employed during ERCP. Many studies have shown that WGC with a standard-tip papillotome (ST-papillotome) facilitates biliary cannulation and reduces PEP compared with CGC [10–13], and WGC with an ST-papillotome has become the first-line cannulation technique worldwide [14–16]. However, even if WGC is done with an ST-papillotome, avoidance of PEP is still challenging, because not only unintended contrast injection but also guide wire insertion into the pancreatic duct can cause PEP [6, 7]. In addition, a prospective trial performed in Japan did not show superiority of WGC with an ST-papillotome over CGC with an ST-papillotome or conventional catheter for achieving successful biliary cannulation and preventing PEP [17]. Therefore, the CGC technique is still widely used for biliary cannulation in Japan. Moreover, Mariani et al. compared the outcomes of WGC or CGC and reported that the excessive manipulation of the main pancreatic duct (e.g., during multiple cannulation attempts) is a more important risk factor for PEP than the actual cannulation technique itself [18]. Thus, the superiority of WGC over CGC may still be somewhat controversial, although WGC with an ST-papillotome is a standard technique for biliary cannulation in the world [14–16].

In order to reduce unintended pancreatic access and facilitate biliary cannulation, we developed a novel oblique-tip papillotome (OT-papillotome). We previously reported that the use of the OT-papillotome reduces unintended contrast injection into the pancreatic duct during CGC compared with the use of a conventional catheter [19]. Thus, it is possible that better results could be obtained by using the OT-papillotome for both CGC and WGC than those reported so far. However, only limited data are available with regard to the utility of this papillotome for biliary cannulation.

Accordingly, this study was performed to evaluate the outcome of the cannulation using the OT-papillotome with CGC or WGC, compared to the use of the ST-papillotome with WGC, which is the current standard cannulation method.

## 2. Materials and Methods

### 2.1. Study Design

This prospective cohort study was performed at two centers: Jikei University Hospital (Tokyo, Japan) and the University of Malaya Medical Centre (UMMC; Kuala Lumpur, Malaysia). Patients who met the eligibility criteria at each hospital between July 2012 and January 2014 were prospectively enrolled. Patients at Jikei University Hospital underwent ERCP by CGC with the OT-papillotome, while patients at UMMC received ERCP by WGC with the OT-papillotome or ST-papillotome. The results obtained at two centers were retrospectively integrated and analyzed. Although randomization to CGC or WGC was carried out at each center in most of the previous comparisons between these techniques [10–13], few centers are expert in both methods and there is usually a preferred first-line technique for biliary cannulation (either WGC or CGC). Since Jikei University Hospital conventionally uses the CGC method and UMMC conventionally uses the WGC method, from the aspect of ethics, the elimination of skill bias and because it takes a long time to obtain stable results with each method, we considered that random allocation was inappropriate for this study. In a study performed by Gray et al. [20], either transcatheter or surgical treatment for patent ductus arteriosus was carried out at multiple hospitals and the results were compared; although all of the patients who met the eligibility criteria were enrolled in the study without random allocation, the good comparability was ensured. We adopted a similar design for the present study. Although Gray et al. performed a retrospective cohort study, we decided to perform a prospective study using a coarsened exact matching (CEM) to minimize bias related to nonrandomized and center-based patient allocation [21]. In short, we did not perform random allocation of the subjects and cannulation was performed by the usual technique at each participating hospital (CGC or WGC). CGC was done with the OT-papillotome (OT-CGC group) at Jikei University Hospital, while WGC was performed with the OT-papillotome or ST-papillotome (OT-WGC group and ST-WGC group, respectively) at UMMC. Data were collected prospectively, and the outcomes of OT-CGC and OT-WGC were retrospectively compared with those of ST-WGC, which is generally regarded as the first-line cannulation technique [14–16]. In addition, same comparison was performed between OT-CGC and OT-WGC.

Inclusion criteria were (1) patients undergoing endoscopic retrograde cholangiography or ERCP-related procedures (e.g., bile sampling, bile duct biopsy, lithectomy, or biliary drainage), (2) patients aged 20 years or older, and (3) patients who could provide written informed consent to participation in this study. Exclusion criteria were (1) patients with a history of ERCP-related procedures, (2) patients with a history of abdominal surgery and Billroth II or Roux-en-Y reconstruction, (3) patients scheduled for pancreatography, (4) patients with serious cardiopulmonary disease or shock making endoscopy difficult, and (5) pregnant or possibly pregnant women. The study protocol was approved by our institutional review board, and the study was registered with the University Medical Information Network (UMIN) (registration number: 000036657).

### 2.2. Concept of the Oblique-Tip Papillotome

**Figure 1:**
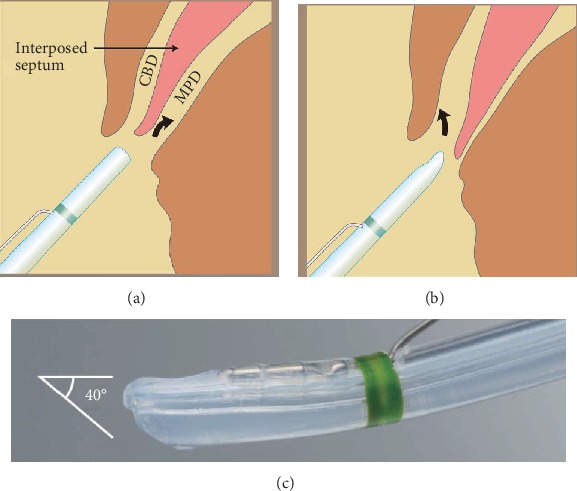
Concept of a new oblique-tip papillotome. A standard-tip papillotome may be advertently aligned with the pancreatic duct (a). The new oblique-tip papillotome can deflect the interposed septum between the opening of the common bile duct and the pancreatic duct below. As a result, the top of the oblique-tip papillotome can follow a course toward the bile duct (b). The new oblique-tip papillotome with cutting wire, injection, and guide wire lumen. The tip of the oblique-tip papillotome is sharpened obliquely and circumferentially and is angled at approximately 40 degrees (c). CBD: common bile duct; MPD: main pancreatic duct.

At the papilla, the opening of the bile duct is usually separated from that of the pancreatic duct by a septum and is located above the pancreatic duct [22]. To selectively cannulate the bile duct, the catheter tip or guide wire should be advanced into the bile duct above the septum by aiming at the 11 o'clock position [23]. Endoscopists sometimes encounter patients in whom cannulation of the pancreatic duct is easy, but deep access to the bile duct remains elusive. In such patients, the tip of the catheter or guide wire may be easy to advance below the septum, i.e., in the direction of the pancreatic duct (Figure 1(a)). Based on this concept, we designed a novel 4.4 Fr oblique-tip papillotome to facilitate biliary cannulation and reduce unintended contrast injection or guide wire insertion into the pancreatic duct. The tip of this new papillotome was angled obliquely and circumferentially and is angled at approximately 40 degrees so that the axis of the tip is more closely aligned with the axis of the bile duct. Therefore, the modified tip can deflect the interposed septum downward and align with the axis of the bile duct, allowing this new papillotome to preferentially enter the bile duct (Figures 1(b) and 1(c)).

### 2.3. ERCP and Cannulation Procedure

#### 2.3.1. Contrast-Guided Cannulation (CGC) with the OT-Papillotome

Two endoscopists with ≥ 10 years of experience, who performed more than 300 ERCPs annually, performed all ERCP procedures at Jikei University Hospital as the operator or supervisor. A side-viewing duodenoscope (TJF-260V, JF-260V; Olympus Medical Systems, Tokyo, Japan) was inserted into the second part of the duodenum, and the papilla of Vater was visualized in the en face view. Then, cannulation was attempted by using the OT-papillotome (KD-VC433Q-0720; Olympus Medical Systems, Tokyo, Japan) with the CGC technique. A small volume of contrast medium was slowly injected to confirm cannulation of the bile duct, after which the OT-papillotome was inserted deeply into the bile duct under fluoroscopic guidance. If injection of contrast medium into the pancreatic duct occurred inadvertently, the catheter was immediately withdrawn from the duct and biliary cannulation was attempted again. If the operator was unable to accomplish deep cannulation of the common bile duct after 10 minutes, the papilla precut technique and/or pancreatic duct guide wire technique was used for cannulation. After deep cannulation was achieved, endoscopic sphincterotomy (EST), endoscopic lithectomy, or endoscopic biliary drainage (EBD) was performed as required.

#### 2.3.2. Wire-Guided Cannulation with the OT-Papillotome or ST-Papillotome

Two endoscopists with ≥ 10 years of experience, who performed annually more than 300 ERCPs annually, performed all ERCP procedures at UMMC as the operator or supervisor. A side-viewing duodenoscope (TJF-Q180V; Olympus Medical Systems, Tokyo, Japan) was inserted into the second part of the duodenum, and the papilla of Vater was visualized in the en face view. Cannulation of the bile duct was performed using an ST-papillotome (Clever Cut 3V: KD-V411M-0730; Olympus Medical Systems, Tokyo, Japan) or the OT-papillotome. After minimal insertion (1-3 mm) of the papillotome across the papilla, a 0.025-inch guide wire was advanced under fluoroscopic guidance. If the guide wire was fluoroscopically confirmed to have entered the bile duct, the papillotome was advanced deeply into the duct. If the guide wire was accidentally inserted into the pancreatic duct, it was immediately withdrawn from the duct and biliary cannulation was attempted again. If the operator was unable to accomplish deep cannulation of the bile duct after 10 minutes, the papilla precut technique and/or pancreatic duct guide wire technique was used for cannulation. After deep biliary cannulation was achieved, EST, endoscopic lithectomy, or EBD was performed as required.

### 2.4. Outcome Measurements and Definitions

The primary endpoint was the number of unintended pancreatic access events (contrast injection or guide wire entry into the pancreatic duct) before successful biliary cannulation. The secondary endpoints were the successful biliary cannulation rate, the biliary cannulation time, the frequency of rescue cannulation, and the incidence of PEP.

The number of unintended pancreatic access events was the total number of times any volume of contrast medium was injected or guide wire entered into the pancreatic duct. Successful biliary cannulation was defined as free and deep instrumentation of the biliary tree. The biliary cannulation time was measured from the time when the papillotome or guide wire first touched the papilla until successful biliary cannulation was achieved, as defined above. Rescue cannulation was defined as the biliary cannulation technique employed when access to the bile duct was not obtained within 10 minutes by WGC or CGC, including precut and pancreatic duct guide wire placement techniques. PEP was diagnosed by the criteria of Cotton et al. [24].

### 2.5. Statistical Analysis

Based on our pilot study [19], sample size calculation indicated that eighty patients per group were required to detect a difference of 1.0 times of unintended pancreatic access event between the OT- papillotome and ST-papillotome cohorts (*α* = 0.05, *β* = 0.8; 2-tailed test).

CEM was performed to adjust for differences of patient factors between the OT-papillotome and ST-papillotome cohorts. CEM is similar to propensity score matching in that it facilitates more comparable evaluation of study groups by creating proportionality among variables that are hypothesized to affect the outcome of interest. Rather than performing matching based on the logit score, CEM divides subjects into distinct strata and they are matched according to relevant variables. Then, the matched subjects are assigned a specific weight for their stratum. CEM has the advantage of being able to balance groups for comparison, while minimizing the confounding effects of individual variables. In addition, it avoids the need for the iterative balance checking process that may introduce errors in propensity score matching, while still maintaining a relative level of similarity between observations [21, 25]. Direct comparison with propensity score matching has shown that the CEM results in less variance and bias. In this study, we matched the OT-papillotome and ST-papillotome cohorts for age (temporarily coarsened into ≤20, 20-29, 30-39,…, 80-89, and ≥90), gender, malignancy of the lesion, and concomitant existence of a diverticulum. We assessed the degree of imbalance between the two datasets before and after matching by measuring multivariate L1 distance. L1 = 0 indicates perfect global balance, and larger values indicate grater imbalance between the cohorts, and L1 = 1 indicates complete separation of the cohorts. Outcomes were estimated by linear, ordered, or logistic regression incorporating CEM weights for continuous, ordinal, or dichotomous variables, respectively.

Variables such as age, biliary cannulation time, and number of unintended pancreatic access events were analyzed with Welch's *t*-test or the Mann–Whitney *U* test. Categorical data, including sex, existence of a diverticulum, underlying diseases, rescue cannulation method, occurrence of PEP, and successful biliary cannulation, were analyzed by the chi-squared test or Fisher's exact probability test.

We also performed multivariate analysis to determine independent association of use of the OT-papillotome with biliary cannulation time, number of unintended pancreatic access events, frequency of rescue cannulation, incidence of PEP, and successful biliary cannulation rate. In addition to the type of papillotome (OT-papillotome vs. ST-papillotome) and the cannulation technique (WGC vs. CGC), the variables used for CEM were included in the multivariate regression models.

Stata statistical software (version14.0; StataCorp LP, College Station, Texas) was used for all analysis, and *p* < 0.05 was considered to indicate statistical significance.

## 3. Results

### 3.1. Patient Characteristics and Cannulation Outcomes in the Unmatched Cohort

Eighty consecutive patients who met the eligibility criteria were enrolled in the OT-CGC group (Jikei University Hospital), while 80 consecutive patients meeting eligibility criteria were enrolled in the OT-WGC group (UMMC) and 80 consecutive patients were enrolled in the ST-WGC group (UMMC). At UMMC, the first 80 patients were treated using the OT-papillotome (OT-WGC group) and the next 80 patients were treated using the ST-papillotome (ST-WGC group).  Table 1 shows patient characteristics before performing CEM. The OT-CGC group was significantly older compared with the ST-WGC group, and the frequency of malignant pancreaticobiliary disease was significantly higher in the OT-CGC group than in the ST-WGC group. Regarding the ERCP procedure, diagnostic ERC (cholangiography), endoscopic biliary drainage, and endoscopic sphincterotomy followed by stone removal were, respectively, performed in 15, 53, and 12 patients from the OT-CGC group; 1, 20, and 59 patients from the OT-WGC group; and 18, 27, and 35 patients from the ST-WGC group. The diagnostic ERC rate was significantly lower in the OT-WGC group than in the ST-WGC group. There was no significant difference of the sex ratio or the presence of a periampullary diverticulum between the ST-WGC group and the OT-CGC group or the OT-WGC group.

The outcomes of biliary cannulation are shown in Table 2. There was no significant difference in the mean number of unintended pancreatic access events between the OT-CGC and ST-WGC groups (1.235 ± 1.925 vs. 0.825 ± 1.581 times, *p* = 0.136) or between the OT-WGC and ST-WGC groups (1.163 ± 1.831 vs. 0.825 ± 1.581, *p* = 0.214). The mean biliary cannulation time was significantly shorter in the OT-CGC group than in the ST-WGC group (318.353 ± 535.386 vs. 490.691 ± 514.384 seconds, *p* = 0.046), although there was no significant difference between the OT-WGC and ST-WGC groups (434.684 ± 486.727 vs. 490.691 ± 514.384 seconds, *p* = 0.499). Compared with the ST-WGC group, the frequency of performing rescue cannulation to achieve successful biliary cannulation was significantly lower in both the OT-CGC group (13.75 vs. 36.25%, *p* = 0.001) and the OT-WGC group (20 vs. 36.25%, *p* = 0.022). Moreover, the successful biliary cannulation rate was significantly higher in both the OT-CGC group (97.5 vs. 83.75%, *p* = 0.002) and the OT-WGC group (98.75 vs. 83.75, *p* = 0.002) than in the ST-WGC group. There was no significant difference in the incidence of PEP between the OT-CGC and ST-WGC groups (5 vs. 6.25%, *p* = 1.0) or between the OT-WGC and ST-WGC groups (5 vs. 6.25%, *p* = 1.0). Comparison between the OT-CGC and OT-WGC groups revealed no significant differences in the mean number of unintended pancreatic access events, mean biliary cannulation time, frequency of rescue cannulation, successful biliary cannulation rate, and incidence of PEP. In all three cohorts, there were no adverse events apart from PEP.

### 3.2. OT-CGC Group vs. ST-WGC Group after CEM (Table 3)

After CEM was performed for the age, gender, malignancy, and concomitant periampullary diverticulum, 75 matches remained in the OT-CGC group and 68 matches in the ST-WGC group. The multivariate L1 distance between the OT-CGC and ST-WGC groups decreased from 0.43 to 0.24, indicating improvement of the imbalance between the groups. The outcomes of biliary cannulation were compared between the OT-CGC and ST-WGC groups. There were no significant differences in the mean number of unintended pancreatic access events (1.26 ± 1.92 vs. 0.88 ± 1.73 times, *p* = 0.063), the mean biliary cannulation time (321.9 ± 539.7 vs. 488.1 ± 534.2 seconds, *p* = 0.092), and the incidence of PEP (5.3 vs. 5.8%, *p* = 0.904). However, the frequency of rescue cannulation was significantly lower in the OT-CGC group than in the ST-WGC group (18.7 vs. 42.3%, *p* = 0.002), and the successful biliary cannulation rate was significantly higher in the OT-CGC group compared with the ST-WGC group (98.7 vs. 74.6%, *p* = 0.002).

### 3.3. OT-WGC vs. ST-WGC after CEM (Table 4)

After CEM was performed for the age, gender, malignancy, and concomitant periampullary diverticulum, 75 matches remained in the OT-WGC group and 73 matches in the ST-WGC group. The multivariate L1 distance between the OT-WGC and ST-WGC groups decreased from 0.25 to 0.108, showing improvement of the between-group imbalance. When the outcomes of biliary cannulation were compared between the OT-WGC and ST-WGC groups, there was no significant difference in the mean number of unintended pancreatic access events (1.24 ± 1.87 vs. 0.76 ± 1.46 times, *p* = 0.084), the mean biliary cannulation time (450.6 ± 494.2 vs. 596.6 ± 624.1 seconds, *p* = 0.133), and the incidence of PEP (4 vs. 6.2%, *p* = 0.546). However, the frequency of rescue cannulation was significantly lower in the OT-WGC group compared with the ST-WGC group (21.3 vs. 44.1%, *p* = 0.004), and the successful biliary cannulation rate was significantly higher in the OT-WGC group than in the ST-WGC group (100 vs. 80.8%, *p* = 0.002).

### 3.4. OT-WGC Group vs. OT-CGC Group after CEM (Table 5)

Biliary cannulation outcomes were also compared between the OT-CGC and OT-WGC groups. After CEM was done for the age, gender, malignancy, and concomitant periampullary diverticulum, 74 matches remained in the OT-CGC group and there were 67 matches in the OT-WGC group. The multivariate L1 distance between the two groups decreased from 0.41 to 0.13, indicating the improvement of the imbalance. There was no significant difference between the OT-CGC and OT-WGC groups with regard to the mean biliary cannulation time, frequency of rescue cannulation, successful cannulation rate, and incidence of PEP. However, the mean number of unintended pancreatic access events was significantly lower in the OT-WGC group than in the OT-CGC group (1.24 ± 1.91 vs. 1.89 ± 2.28 times, *p* = 0.038).

### 3.5. Multivariate Analysis

Multivariate analysis revealed a significant negative correlation between the use of the OT-papillotome and the frequency of rescue cannulation (OR: 0.447, *p* = 0.028, 95% CI: 0.218-0.916), as well as a significant positive correlation with the successful biliary cannulation rate (OR: 21.906, *p* = 0.006, 95% CI: 2.401-199.864). However, there was no independent association of OT-papillotome use with the biliary cannulation time, the number of unintended pancreatic access events, or the incidence of PEP (Table 6).

## 4. Discussion

PEP is the most common and severe complication of ERCP, with the reported incidence ranging from 1.3% to 15.1% in prospective studies [1–7]. A number of risk factors have been reported to show a significant association with PEP, including young age, female sex, sphincter of Oddi dysfunction, a history of PEP, unintended pancreatic access (contrast injection or guide wire entry), and difficult cannulation [1–7]. Theoretically, it might be possible to eliminate all of the cannulation-related risk factors for PEP such as unintended pancreatic access and difficult cannulation. To facilitate biliary cannulation and prevent PEP, WGC with the ST-papillotome was developed as a cannulation technique. Subsequently, many studies comparing WGC and CGC have been conducted [10–16]. A recent meta-analysis of 12 RCTs (3450 patients) by Tse et al. showed that PEP was significantly less frequent and the successful cannulation rate was higher with WGC compared to CGC [13]. The authors recommended WGC as an appropriate first-line cannulation technique for ERCP. However, this meta-analysis only identified a significant reduction in the risk of PEP by WGC in “noncrossover” trials. In addition, WGC was not associated with a higher successful biliary cannulation rate or lower incidence of PEP in 3 RCTs performed in Japan [17, 26, 27]. It seems likely that these Japanese trials did not show superiority of WGC over CGC because a 15-degree backward-oblique angle duodenoscope (BOAD) is widely used for ERCP in Japan, which facilitates adjusting the direction of cannulation to the axis of the bile duct compared with the 5-degree BOAD used in previous RCTs [28, 29]. In addition, “crossover” trials [13, 18] reported that the risk of PEP was similar with WGC or CGC. They found that the significant risk factors independently associated with PEP were more than 10 cannulation attempts, pancreatic access, sphincter of Oddi dysfunction, and use of the precut procedure, and they concluded that manipulation of the main pancreatic duct was associated with a high risk of PEP rather than the cannulation techniques. We also hypothesized that biliary cannulation without causing trauma to the papilla or unintended access of pancreatic duct may be more important for preventing PEP than the choice of WGC or CGC. Based on this hypothesis, we developed the OT-papillotome to facilitate biliary cannulation and reduce unintended pancreatic access events.

To verify our hypothesis about the utility of the OT-papillotome, we conducted the present comparison of OT-CGC and OT-WGC with ST-WGC, which is currently a standard cannulation method worldwide [14–16]. Our study design had several limitations because of patient allocation being nonrandomized and center-based. However, this method has already been reported by Gray et al. [20], and they achieved good comparability. Although most of the previous comparisons of CGC with WGC have been randomized studies [10–13], few centers are expert in both cannulation methods. That is, most centers have a preferred first-line cannulation method and the operators at a particular center are more familiar with either WGC or CGC. Therefore, comparison of CGC with WGC is almost invariably affected by skill bias. The nonrandomized and center-based design for patient allocation employed in this study might have the advantage of minimizing skill bias because each hospital managed patients by its standard method. Moreover, we used CEM to eliminate bias due to nonrandomized and center-based patient allocation. CEM is a relatively new matching method of the monotonic imbalance bounding class [30]. When CEM is used, reducing the imbalance in the empirical distribution of one covariate has no effect on any other covariate chosen for balancing, which represents a clear advantage over other matching methods, including propensity score matching. For balance checking, the multivariate imbalance measure L1 was introduced [21]. In this study, the imbalance between the OT-CGC and ST-WGC groups, OT-WGC and ST-WGC groups, and OT-WGC and OT-CGC groups measured by the multivariate L1 distance improved to 0.24, 0.108, and 0.13, respectively.

After CEM was performed, the successful biliary cannulation rate was significantly higher in both the OT-CGC and OT-WGC groups than the ST-WGC group. In addition, the frequency of rescue cannulation was significantly lower in both the OT-CGC and OT-WGC groups than the ST-WGC group. However, the mean number of unintended pancreatic access events and the incidence of PEP in both the OT-CGC and OT-WGC groups were similar with those in the ST-WGC group. Multivariate analysis revealed that use of the OT-papillotome was independently associated with successful biliary cannulation and less frequent rescue cannulation. Thus, the OT-papillotome could facilitate biliary cannulation by both CGC and WGC, while reducing the need for rescue technique compared with the combination of the ST-papillotome and WGC (i.e., standard cannulation technique).

We found that the mean number of unintended pancreatic access events was significantly lower in the OT-WGC group than the OT-CGC group, although there were no significant differences with regard to the successful biliary cannulation rate, the frequency of rescue cannulation, and the incidence of PEP. These findings suggest that WGC with the OT-papillotome might be the most appropriate first-line cannulation technique for ERCP.

Finally, compared to the ST-papillotome, the OT-papillotome did not reduce the rate of PEP despite improving the successful biliary cannulation rate. However, the possibility that the OT-papillotome could reduce the incidence of PEP remains, due to the elimination of one of the cannulation-related risk factors of PEP—biliary cannulation. Therefore, a larger study is needed to clarify the utility of the OT-papillotome in the prevention of PEP.

This study had several limitations. It was nonrandomized, and center-based patient allocation was performed, with only two centers being involved. Accordingly, a prospective multicenter study with more centers is needed to further clarify the utility of the OT-papillotome for ERCP.

## 5. Conclusion

Compared to the ST-papillotome, the OT-papillotome increased the rate of successful biliary cannulation with both CGC and WGC (compared to ST-WGC), while reducing the frequency of rescue cannulation procedures. However, the OT-papillotome-facilitated biliary cannulation did not reduce unintended pancreatic access events or PEP. Combining the OT-papillotome with WGC might be the most favorable cannulation technique for minimizing unintended pancreatic access.

## Figures and Tables

**Table 1:** Patient characteristics in the unmatched cohort.

	OT-CGC	OT-WGC	ST-WGC
Number of patients	80	80	80
Age	67.793 ± 12.102^1,4^	58.65 ± 17.979	58.086 ± 18.926
Sex (male/female)	48/32	36/44	37/43
Underlying disease (benign/malignant)	47/33^2,5^	69/11	65/15
Choledocolithiasis	41	57	51
Other benign disease	6	12	14
Pancreatic cancer	10	3	7
Biliary cancer	14	6	8
Other malignancy	9	2	0
Diverticulum (-/+)	70/10	76/4	77/3
Endoscopic procedure (ERC/EBD/EST)	15/53/12	1/20/59^3^	18/27/35

ERC: endoscopic retrograde cholangiography; EBD: endoscopic biliary drainage; EST: endoscopic sphincterotomy. ^1^*p* = 0.0001 (OT-CGC vs. ST-WGC), ^2^*p* = 0.0019 (OT-CGC vs. ST-WGC), ^3^*p* = 0.001 (OT-WGC vs. ST-WGC), ^4^*p* = 0.0001 (OT-CGC vs. OT-WGC), and ^5^*p* = 0.0002 (OT-CGC vs. OT-WGC).

**Table 2:** Outcomes of biliary cannulation in the unmatched cohort.

	OT-CGC	*p*	OT-WGC	*p*	ST-WGC
Number of unintended pancreatic access events	1.235 ± 1.925	0.135	1.162 ± 1.831	0.214	0.825 ± 1.581
Biliary cannulation time (seconds)	318.353 ± 535.386	0.045	434.684 ± 486.727	0.499	490.691 ± 514.384
Frequency of rescue cannulation (%)	13.75 (11/80)	0.001	20 (16/80)	0.022	36.25 (29/80)
Successful biliary cannulation rate (%)	97.5 (78/80)	0.002	98.75 (79/1)	0.002	83.75 (67/80)
Incidence of PEP (%)	5 (4/80)	1.0	5 (4/80)	1.0	6.25 (5/80)

**Table 3:** Comparison of cannulation outcomes in the OT-CGC and ST-WGC groups by coarsened exact matching.

	OT-CGC (*n* = 75)	ST-WGC (*n* = 68)	OR or coefficient (95% CI)	*p*	Statistical method
Number of unintended pancreatic access	1.26 ± 1.92	0.88 ± 1.73	1.94 (0.966-3.884)	0.063	Ordinal logistic regression
Time to biliary cannulation (second)	321.9 ± 539.7	488.1 ± 534.2	-166.2 (-360.212-27.750)	0.092	Linear regression
Frequency of the use of rescue cannulation method (%)	18.7% (13/75)	42.3% (29/68)	0.29 (0.135-0.627)	0.002	Logistic regression
Successful biliary cannulation rate (%)	98.7% (74/75)	74.6% (51/68)	25.13 (3.24-194.732)	0.002	Logistic regression
Incidence of PEP (%)	5.3% (4/75)	5.8% (4/68)	0.92 (0.219-3.830)	0.904	Logistic regression

**Table 4:** Comparison of cannulation outcomes in the OT-WGC and ST-WGC groups by coarsened exact matching.

	OT-WGC (*n* = 75)	ST-WGC (*n* = 73)	OR or coefficient (95% CI)	*p*	Statistical method
Number of unintended pancreatic access events	1.24 ± 1.87	0.76 ± 1.46	1.80 (0.924-3.496)	0.084	Ordinal logistic regression
Biliary cannulation time (seconds)	450.6 ± 494.2	596.6 ± 624.1	-145.9 (-337.064-45.123)	0.133	Linear regression
Frequency of rescue cannulation (%)	21.3% (16/75)	44.1% (32/73)	0.34 (0.167-0.706)	0.004	Logistic regression
Successful biliary cannulation rate (%)	100% (75/75)	80.8% (59/73)	N/A	0.002	Chi-square
Incidence of PEP (%)	4% (3/75)	6.2% (4.5/73)	0.63 (0.141-2.817)	0.546	Logistic regression

**Table 5:** Comparison of cannulation outcomes in the OT-WGC and OT-CGC groups by coarsened exact matching.

	OT-WGC (*n* = 67)	OT-CGC (*n* = 74)	OR or coefficient (95% CI)	*p*	Statistical method
Number of unintended pancreatic access events	1.24 ± 1.91	1.89 ± 2.28	0.514 (0.274-0.165)	0.038	Ordinal logistic regression
Biliary cannulation time (seconds)	457.9 ± 509.0	344.0 ± 513.1	113.8 (-58.657-286.356)	0.194	Linear regression
Frequency of rescue cannulation (%)	22.4% (15/67)	24.1% (18/74)	0.89 (0.406-1.949)	0.77	Logistic regression
Successful biliary cannulation rate (%)	98.5% (66/67)	96.8% (72/74)	2.21 (0.209-23.305)	0.51	Logistic regression
Incidence of PEP (%)	3% (2/67)	8.7% (6/74)	0.31 (0.062-1.597)	0.163	Logistic regression

**Table 6:** Multivariate regression models: factors associated with the biliary cannulation time, number of unintended pancreatic access events, frequency of rescue cannulation, biliary cannulation rate, and incidence of PEP.

	Coefficient or odds ratio (95% CI)	*p* value
Objective variable: number of unintended pancreatic access events		
OT-papillotome (vs. ST-papillotome)	1.579 (0.825-3.023)	0.168
Age	1.003 (0.987-1.019)	0.735
Male (vs. female)	0.555 (0.328-0.9388)	0.028
Malignancy (vs. benign disease)	0.783 (0.403-1.522)	0.470
Diverticulum (vs. no diverticulum)	0.619 (0.22-1.745)	0.364
Wire-guided cannulation (vs. contrast-guided)	0.781 (0.408-1.499)	0.458
Objective variable: biliary cannulation time		
OT-papillotome (vs. ST-papillotome)	-62.915 (-231.882-106.053)	0.464
Age	1.779 (-2.435-5.994)	0.406
Male (vs. female)	-18.156 (-154.931-118.618)	0.794
Malignancy (vs. benign disease)	54.622 (-115.241-224.484)	0.527
Diverticulum (vs. no diverticulum)	-138.70 (-414.775-137.376)	0.323
Wire-guided cannulation (vs. contrast-guided)	131.248 (-40.312-302.807)	0.133
Objective variable: frequency of rescue cannulation		
OT-papillotome (vs. ST-papillotome)	0.447 (0.2185-0.916)	0.028
Age	0.991 (0.973-1.009)	0.311
Male (vs. female)	0.850 (0.462-1.564)	0.601
Malignancy (vs. benign disease)	1.596 (0.760-3.354)	0.217
Diverticulum (vs. no diverticulum)	1.079 (0.321-3.627)	0.902
Wire-guided cannulation (vs. contrast-guided)	1.127 (0.491-2.583)	0.778
Objective variable: incidence of PEP		
OT-papillotome (vs. ST-papillotome)	0.791 (0.203-3.080)	0.735
Age	0.992 (0.959-1.026)	0.633
Male (vs. female)	0.486 (0.153-1.550)	0.223
Malignancy (vs. benign disease)	1.471 (0.385-5.622)	0.572
Diverticulum (vs. no diverticulum)	0.984 (0.110-8.839)	0.989
Wire-guided cannulation (vs. contrast-guided)	0.779 (0.180-3.361)	0.738
Objective variable: successful biliary cannulation		
OT-papillotome (vs. ST-papillotome)	21.906 (2.401-199.864)	0.006
Age	1.002 (0.966-1.040)	0.895
Male (vs. female)	3.318 (0.904-12.178)	0.071
Malignant (vs. benign disease)	0.253 (0.061-1.046)	0.058
Diverticulum (vs. no diverticulum)	0.056 (0.008-0.408)	0.004
Wire-guided cannulation (vs. contrast-guided)	0.924 (0.071-12.097)	0.952

## Data Availability

The data used to support the findings of this study are restricted by the institutional review board of The Jikei University School of Medicine and University of Malaya Medical Centre, in order to protect the patient privacy.
